# Evaluation of *SHOX *copy number variations in patients with Müllerian aplasia

**DOI:** 10.1186/1750-1172-6-53

**Published:** 2011-08-02

**Authors:** Maria Sandbacka, Mervi Halttunen, Varpu Jokimaa, Kristiina Aittomäki, Hannele Laivuori

**Affiliations:** 1 Folkhälsan Institute of Genetics, Helsinki, Finland; 2 Department of Medical Genetics, Haartman Institute, University of Helsinki, Helsinki, Finland; 3 Department of Obstetrics and Gynecology, Helsinki University Central Hospital, Helsinki, Finland; 4 Department of Obstetrics and Gynecology, University of Turku, Turku, Finland; 5 Department of Clinical Genetics, Helsinki University Central Hospital, Helsinki, Finland; 6 Research Programs Unit, Women's Health, University of Helsinki, Helsinki, Finland

## Abstract

**Background:**

Müllerian aplasia (MA) characterized by congenital loss of functional uterus and vagina is one of the most difficult disorders of female reproductive health. Despite of growing interest in this research field, the cause of the disorder for the majority of patients is still unknown. A recent report of partial *SHOX *duplications in five patients with MA has motivated us to further evaluate their role in the disorder. Therefore we have studied *SHOX *copy number variations (CNVs) in a cohort of 101 Finnish patients with MA and in 115 healthy controls.

**Methods:**

We used multiplex ligation-dependent probe amplification (MLPA) to study *SHOX *CNVs.

**Results:**

All patients showed normal amplification of *SHOX*. Several aberrations, duplications and deletions, were found downstream of the gene in five patients and seven controls, but these were all copy number polymorphisms.

**Conclusions:**

Our study in an extensive cohort of patients with MA does not support a role for *SHOX *CNVs in the aetiology of the disorder. Further studies in the field are important for both patients looking for answers as well as for the scientific community for better understanding the regulation of the female reproductive duct development.

## Background

The Müllerian ducts form the primordial basis for the female reproductive tract. They differentiate into the oviducts, uterus, and the upper two-thirds of the vagina during early embryonic development. A wide variety of malformations can occur if this development is disrupted. One such malformation is Müllerian aplasia (MA), also referred to as Mayer-Rokitansky-Küster-Hauser (MRKH, Online Mendelian Inheritance in Man [OMIM] #277000) syndrome or MURCS assosciation (Müllerian duct aplasia, Renal dysplasia and Cervical Somite anomalies, OMIM #601076) as renal and skeletal malformations are relatively common in patients with MA [1]. Women with MA are otherwise healthy with normal female chromosome constitution (46, XX) and normal secondary sexual characteristics. MA is commonly diagnosed in young adulthood due to primary amenorrhoea. The effects on sexual life with infertility often cause lifelong psychosocial problems making MA one of the most difficult disorders of female reproductive health. The minimum incidence of MA is 1 in 5000 newborn girls [2], and for the majority of the patients the cause is still unknown.

During recent years, an increasing number of studies have aimed at investigating the genetic basis of MA. To date, only mutations in *WNT4 *(the wingless-type MMTV integration site family, member 4 gene) have been reported to cause MA [3-6]. However, the phenotype of the four patients with *WNT4 *mutations includes hyperandrogenism, which usually is not associated with MA, suggesting that these patients form a distinct subclass of MA (OMIM #158330). Several other candidate genes involved in fetal development and sex differentiation have been thoroughly investigated without further success as reviewed by Sultan and colleagues [7].

Chromosomal imbalances studied by array comparative genomic hybridization (aCGH) have been reported for several chromosomal areas in MA patients [8-14]. However, as these are located in several different locations along the genome (1q21.1, 16p11.2, 17q12, 22q11.2 and Xq21.31), they have not revealed strong candidate regions or genes for MA. Interestingly, partial duplications of *SHOX *(short stature homeobox gene) were recently reported in five patients (two sporadic, three familial) with MA [15]. In one family, the same duplication was present in two sisters with MA and their healthy father, but absent in two healthy sisters and their mother suggesting a dominant inheritance of MA from an unaffected male carrier.

*SHOX *is a homeobox gene covering 40 kb on the pseudoautosomal region (PAR1) of the X (Xp22) and Y (Yp11.3) chromosomes. Like other genes within the PAR1, *SHOX *escapes X inactivation and therefore both alleles are normally expressed in males and females. Mutations and deletions in *SHOX *have been reported to cause short stature in patients with idiopathic short stature (ISS, OMIM #300582), Turner syndrome [16,17], dyschondrosteosis (Leri-Weill syndrome, LWD, OMIM #127300) [18] and its more severe form Langer mesomelic dysplasia (OMIM #249700) [19]. Recently, some patients with ISS and LWD have been reported to carry duplications of *SHOX*/PAR1 region [20].

To further evaluate the role of *SHOX *in MA, we have studied *SHOX *with MLPA (multiplex ligation-dependent probe amplification) in a cohort of 101 Finnish patients with MA and 115 healthy female controls.

## Patients and methods

### Patients

Patients with MA were recruited to the study through the Departments of Obstetrics and Gynecology of the five University Hospitals in Finland from year 1978 onwards. The cohort included 101 Finnish patients, of whom two are siblings. The clinical phenotype of all patients includes congenital loss of the uterus and the upper two-thirds of the vagina, while the status of the oviducts is not known for all patients. The patients were otherwise healthy females with a normal female chromosome constitution, hormonally active functioning ovaries, and normal secondary sexual characteristics. One hundred and fifteen women with at least one normal pregnancy served as controls. Informed consent was obtained from all patients and controls before recruitment. The study protocol has been approved by the Ethics Committee of the Department of Obstetrics and Gynecology, Helsinki University Central Hospital, Finland, and the Finnish Ministry of Social Affairs and Health.

### Sample preparation

DNA from the patients and controls was extracted from peripheral blood samples using the Puregene DNA Isolation Kit (Gentra Systems, Minneapolis, MN, USA), or by the phenol-chloroform method. The quality and quantity of DNA was analyzed by NanoDrop ND-1000 spectrophotometer (Thermo Fisher Scientific, Waltham, MA, USA).

### Multiplex ligation-dependent probe amplification (MLPA)

The commercial SALSA MLPA kit PO18-E1 SHOX (MRC-Holland, Amsterdam, Netherlands) was used for the amplification reactions according to the manufacturer's recommendation. The MLPA mix included probes for each exon of *SHOX*, one probe just before the promoter region as well as probes covering a region downstream of the gene. In short, 100 or 200 ng of DNA was denatured and incubated with the MLPA probes for 16-18 h in 60°C. The probes were ligated to the DNA and amplified by PCR. Thereafter, the PCR products were visualized on an agarose gel (1.5%, Bioline, London, UK), appropriately diluted and combined with 1% formamide (Applied Biosystems, Foster City, CA, USA) and GeneScan™-500 LIZ™ size standard (Applied Biosystems). The products were then separated by capillary electrophoresis on an ABI3730XL DNA Analyzer (Applied Biosystems). The results were analyzed by GeneMapper software version 4.0 (Applied Biosystems) and MRC Coffalyzer MLPA-Dat Software (MRC-Holland). The results were also verified by the following calculation. Each peak area was divided by the sum of all peak areas, and the quotient was further divided by the sum of all peak areas of the reference sample. A reduction or increase of 40% or more in peak area, when compared to normal controls, was taken as suggestive for an aberrant amplification. All aberrant results were confirmed by a second independent MLPA analysis.

## Results

**Figure 1:**
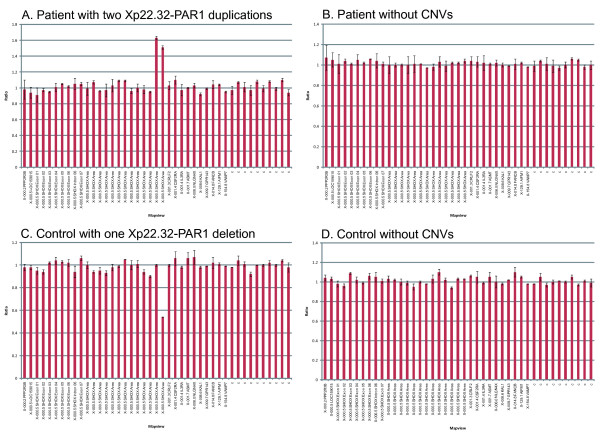
**Examples of the MLPA results**. Four examples of MLPA results illustrating A) a patient with duplication identified with two probes in the Xp22.32-PAR1 region, B) a patient without copy number variations (CNVs), C) a healthy control with a deletion in the Xp22.32-PAR1 region, and D) a healthy control without identified CNVs.

All investigated MA samples (N = 101) showed normal amplification of *SHOX *by MLPA. Aberrant amplification of some probes downstream of *SHOX *was detected in five MA and seven control samples (examples of the MLPA results are presented in Figure 1). One patient sample and one control showed two copy number variations (CNVs), all others samples showed one aberration each. One larger duplication spanning eleven probes from 13296-L15336 in Xp22.32-PAR1 to 13911-L16505 in the *CRLF2 *gene (about 620 kb) was seen in one control sample (Table 1).

**Table 1:** Genomic aberrations found in patients with Müllerian aplasia and in healthy controls.

Region/gene	Aberration	MLPA probe	Cases	Controls	Reference
Xp22.32-PAR1	Gain	09335-L15508^*a*^	1*	1*	DGV^*b*^, Gervasini *et al*, 2010
Xp22.32-PAR1	Loss	09335-L15508	1	1	DGV
Xp22.32-PAR1	Gain	14697-L16348^*a*^	1*	1*	DGV, Gervasini *et al*, 2010
Xp22.32-PAR1	Loss	14697-L16348		1	DGV
Xp22.32-PAR1 to CRLF2	Gain	13296-L15336 -13911-L16505		1	all 11 probes reported in DGV
CSF2RA	Gain	10251-L15502		2	DGV
IL3RA	Gain	13597-L15055	1		DGV
ASMT	Gain	01153-L00712^*c*^	2	1	DGV, Gervasini *et al*, 2010

Genomic aberrations found downstream of *SHOX *(short stature homeobox gene) by multiplex ligation-dependent probe amplification (MLPA, SALSA PO18-E1 SHOX) in 101 Finnish patients with Müllerian aplasia and in 115 healthy controls. One patient sample and one control sample had two copy number variations (CNVs)*, all other samples had one CNV each.

^*a *^the probe sequence is partly overlapping with one MLPA probe (5650-L5104) from version PO18-B SHOX used by Gervasini *et al*, 2010.

^*b *^DGV (Database of Genomic Variants, http://projects.tcag.ca/variation)

^*c *^the probe sequence covers the same genomic region as one MLPA probe (1153-L0712) from version PO18-B SHOX used by Gervasini *et al*, 2010.

## Discussion

The recent findings of partial *SHOX *duplications in three familial and two sporadic cases of MA [15] has motivated us to evaluate their occurrence in a larger patient cohort. We investigated 101 well-characterized Finnish MA patients and 115 controls by the MLPA technique without identifying any *SHOX *aberrations that could be interpreted as causative. We did observe copy number changes in twelve samples (five patients, seven controls) downstream of *SHOX *(Table 1), a region known to be involved in the gene regulation [21,22]. However, these changes were all reported in the Database of Genomic Variants (DGV, http://projects.tcag.ca/variation) and three of them by Gervasini *et al*. [15] as harmless CNVs without phenotypic effect. A duplication 5'of *SHOX *spanning about 620 kb from Xp22.32-PAR1 to the *CRLF2 *gene was found in one control sample. The entire duplication is not reported in DGV, but the genomic sequences of all eleven probes within the duplication are noted as known copy number variants.

In the study by Gervasini *et al*. 3/3 familial cases and 2/27 sporadic cases (7%) of MA showed partial duplications of *SHOX*. The clinical phenotype of these patients fulfills the same criteria as defined in our study cohort: absence of vagina and functional uterus in otherwise healthy females. If the inheritance of *SHOX *duplications in MA is dominant, as suggested, and based on above-mentioned percentages we would expect several patients with *SHOX *duplications in the Finnish cohort (two sisters and seven of the sporadic patients). However, by implementing the same method as Gervasini and coworkers in a more than three times larger patient cohort, we did not find any aberrations in *SHOX *suggesting that *SHOX *duplications are not a major cause of MA. Point mutations in *SHOX *resulting in a gain-of-function situation cannot, however, be excluded by the MLPA method.

Overall, duplications of the *SHOX *gene are rarely described in the literature. *SHOX *is thought to regulate human skeletal growth, and deletions of the gene to result in short stature (as in Turner syndrome) and an extra copy in tall stature (as in sex chromosome trisomies). The first case of *SHOX *duplication, not reported in conjunction with a larger chromosome aberration, was a female with isolated Madelung deformaties of the wrists. Interestingly, the duplication was also present in her healthy sister, making the clinical significance of the duplication unclear [23]. Subsequently, four more reports of patients with *SHOX *duplications were published [20,24-26]. Unfortunately, the status of the uterus and vagina of the patients was not described in these reports. Furthermore, *SHOX *duplication in conjunction with chromosomal deletions on the long arm of X (Xq) has been reported in four females [27-30], one of whom had been reported with premature ovarian failure [30]. The authors suggested that the duplication in this patient could be involved in the ovarian insufficiency. The presence of uterus was described for three of the patients [27,29,30], of whom one had experienced an early spontaneous abortion [30]. Unfortunately the status of uterus and vagina was not described in the fourth patient [28].

## Conclusions

Taken together, there is no clear evidence in the literature for a role for *SHOX *in the development of the female reproductive duct. Secondly, our result based on an extensive series of Finnish patient samples does not support *SHOX *duplications as a frequent cause of MA. Finally, the duplications of *SHOX *found by Gervasini and co-workers [15] in combination with MA can be coincidental and may not be a reflection of an underlying genetic relationship. However, the aetiology of MA points to multifactorial inheritance. Partial duplications of *SHOX *might therefore be one of several genetic causes that contribute to the development of MA. Population genetic differences could explain why no duplications of *SHOX *were found in the Finnish cohort compared to the Italian. Furthermore, the possibility of point mutations in *SHOX *resulting in a gain-of-function situation cannot be excluded in our study.

Further studies aiming at revealing the genetic and molecular background of MA are important for both patients looking for an explanation for their symptoms as well as for the scientific community. Understanding the molecular basis of MA will be critical in increasing our knowledge for the regulation of the female reproductive tract development.

## Competing interests

The authors declare that they have no competing interests.

## Authors' contributions

MS: project design, laboratory work and result analysis, manuscript preparation.

MH: clinician examining and enrolling patients in the study, manuscript preparation.

VJ: clinician examining and enrolling patients in the study, manuscript preparation.

KA: principal investigator, project design, result analysis, manuscript preparation.

HL: senior investigator, project design, result analysis, manuscript preparation.

All authors read and approved the final manuscript.
